# Molecular and Clinical Characterization of a Cohort of Autosomal Recessive Sensorineural Hearing Loss in Egyptian Patients

**DOI:** 10.1007/s12031-024-02279-3

**Published:** 2024-10-28

**Authors:** Mohammed M. Sayed-Ahmed, Hala T. El-Bassyouni, Hanan H. Afifi, Mona L. Essawi, Mohamed B. Taher, Mohamed I. Gadelhak, Rehab A. Zaytoun, Ahmed A. Abdelmonem, Nagham M. Elbagoury

**Affiliations:** 1 Clinical Genetics Department, Human Genetics and Genome Research Institute, National Research Centre, Cairo, Egypt; 2 Medical Molecular Genetics Department, Human Genetics and Genome Research Institute, National Research Centre, Cairo, 12311 Egypt; 3 Phoniatrics Unit, Faculty of Medicine, Fayoum University, Fayoum, Egypt; 4 Phoniatrics Unit, Faculty of Medicine, Beni-Suef University, Beni Suef, Egypt

**Keywords:** WES, MYO7A, *GJB2*, *BSND*, *OTOF*, ARNSHL

## Abstract

Hearing loss (HL) is one of the most common health problems worldwide. Autosomal recessive non-syndromic sensorineural hearing loss (ARNSHL) represents a large portion of congenital hereditary HL. Our study was conducted on 13 patients from 13 unrelated families. The majority of patients presented with congenital severe to profound bilateral sensorineural HL. All patients were subjected to detailed family history and three-generation pedigree analysis to exclude any environmental cause and to ensure an autosomal recessive mode of inheritance. Molecular analysis was performed using the whole exome sequencing (WES) technique for the recruited patients. Three variants in the *MYO7A* and *OTOF* genes were reported for the first time in patients with ARNSHL (one nonsense, one frameshift, and one splice variant). Ten previously reported variants were detected in seven genes (*GJB2*, *MYO15A*, *BSND*, *OTOF*, *CDH23*, *SLC26A4*, and *TMIE*). They varied between missense, nonsense, frameshift, and splice variants. This study expands the molecular spectrum of two types of autosomal recessive deafness (types 2 and 9).

**Supplementary Information:**

The online version contains supplementary material available at 10.1007/s12031-024-02279-3.

## Introduction

Hearing loss (HL) is one of the most common hindering health problems worldwide. In the USA, it is considered the most prevalent sensory disorder (Haile et al. 2024). Prelingual HL has an incidence of 1 in 500 (Delmaghani and El-Amraoui 2020). The World Health Organization (WHO) stated that about 5% of the world’s population (466 million people) is affected by HL and is expected to reach 1 billion by 2050 according to the organization’s report in 2018. In 2021, WHO raised the expected number to 2.5 billion by the year 2050 which reflects the increasing effect of this health problem on international society (https://www.who.int/news-room/fact-sheets/detail/deafness-and-hearing-loss). Causes of HL can be genetic where genetic variations are thought to cause at least 50% of prelingual HL (Friedman and Griffith 2003; Raviv et al. 2010), age-related as about 70% of people over 70 years suffer from age-related HL or environmental due to exposure to environmental stresses including noise, viruses, chemicals, or ototoxic drugs. These stresses can cause permanent sensorineural HL by damaging inner and outer auditory hair cells and neurons (Liberman 2017).

In Egypt, the application of neonatal hearing screening started in 2019. It served in the early detection of hearing impairment and the application of early intervention programs. An incidence of 14% was reported in Egypt (ElGindy et al. 2022). This is comparable to the incidence of (13%) in Saudi Arabia (Zakzouk and Al-Anazy 2002). The incidence was relatively lower in Oman (5.53%) (Khabori et al. 1996) and Jordan (1.5%) (Sidenna et al. 2020).

Despite the burden imposed by HL, current treatments are limited to hearing devices and cochlear implantation which are useful but cannot restore normal levels of hearing (Delmaghani and El-Amraoui 2020). Recently, gene therapy has been adopted as a treatment approach in animal model trials. The afflicted gene, its inheritance mechanism, and occasionally the pathogenic variant determine which strategy should be used.

Exploring the genetic etiology of autosomal recessive non-syndromic sensorineural hearing loss (ARNSHL) could help in accurate genetic counseling for the patients and their families. This can, in turn, limit the incidence of the disease through premarital, preimplantation, and prenatal genetic testing. In this regard, the purpose of this study was to investigate the genetic etiology of a cohort of patients with ARNSHL.

## Patients and Methods

Thirteen patients provisionally diagnosed with ARNSHL were recruited from patients referred to the Clinical Genetics Department, National Research Centre (NRC), and the Phoniatrics Clinics in Beni-Suef and El-Fayoum Universities.

### Clinical Evaluation

Thirteen patients from 13 unrelated families were recruited for this study (9 males and 4 females). Clinical evaluation included complete gestational history-taking to exclude environmental causes of deafness as the exposure of the mother to viruses, drugs, or radiation. A three-generation family pedigree was constructed. In addition, a thorough clinical examination to exclude syndromic deafness was done. Oral examination was important to assess tongue structure and movement as well as tone and power of oral muscles. An ear, nose, and throat (ENT) examination was performed to exclude any ear malformations. Autism spectrum disorders and attention-deficit hyperactivity (ADHD) were excluded using the 5th edition of the *Diagnostic and Statistical Manual of Mental Disorder* (DSM-V) (Edition 2013). Nonverbal intelligent quotient (IQ) testing was performed using the Stanford-Binet Intelligence Scale-5th edition (Roid and Pomplun 2012). This was followed by audiological assessment in the form of auditory brainstem response (ABR) to evaluate the hearing threshold and classify the severity of HL. All parents were counseled regarding the improvement of receptive and expressive language of their children affected with hearing loss.

### Molecular Analysis

The whole exome sequencing (WES) technique was performed with a read depth of 100 × for more than 98% of the targeted bases. The main steps encompass extraction of gDNA and fragmentation of the isolated nucleic acid. This is followed by library preparation, colony formation, and sequencing. The processing of data is done through the usage of bioinformatics tools, and finally, bioinformatics analysis of the output data is carried out. The investigation of related variants was mainly done for coding exons and ten bases flanking region up- and downstream in the intronic regions. All potential patterns for a mode of inheritance were considered.

### In Silico Functional Analysis

Different in silico functional tools were used to predict the pathogenicity of detected variants. The tools specific for splicing variants were dbscSNV, Splice AL, and MaxEntScan. The tools specific for nonsense and missense variants were EIGEN, GenoCanyon, FATHMM-MKL, DANN, EIGEN PC, BayesDel, and LRT.

## Results

The patients included nine males 69.2% (9/13) and four females 30.8% (4/10); their ages ranged from 2 to 12 years. The family pedigree analysis of the patients suggested an autosomal recessive mode of inheritance. Eleven patients were the offspring of consanguineous parents. The families of ten of the patients had similarly affected family members. Twelve patients performed IQ testing, and scores ranged from 80 to 96. Auditory brain stem response (ABR) revealed bilateral severe to profound SNHL in 11 patients, and bilateral moderate SNHL in 2 patients as shown in Table 1.

**Table 1:** The clinical and molecular findings of the patients

ID	Gene	Amino acid change	Zygosity	Age/gender	Consanguinity	Family history	ABR	IQ	Phenotype (OMIM)	Variant reference
1	*MYO7A*	p.?	Homo	11/M	_+_	+	Bilateral severe to profound SNHL	82	Deafness, autosomal recessive 2 (600060)	This study
2	*MYO7A*	Gln1333Ter	Homo	6/M	+	+	Bilateral severe to profound SNHL	80	Deafness, autosomal recessive 2 (600,060)	Budde et al. (2020)
3	*OTOF*	Trp416Ter	Homo	4/M	+	+	Bilateral severe to profound SNHL	88	Deafness, autosomal recessive 9 (601,071)	This study
4	*OTOF*	Ser1240ArgfsTer57	Compound hetero	12/M	+	+	Bilateral severe to profound SNHL	80	Deafness, autosomal recessive 9 (601,071)	This study
		Arg1792Cys								Almontashiri et al. (2018)
5	*MYO15A*	Arg1735Trp	Homo	4/M	+	+	Bilateral severe to profound SNHL	96	Deafness, autosomal recessive 3 (600,316)	Salime et al. (2017)
6	*GJB2*	Gly12ValfsTer2	Homo	2/M	_	+	Bilateral severe to profound SNHL	N/A	Deafness, autosomal recessive 1A (220,290)	Rodriguez-Ballesteros et al. (2008)
7	*GJB2*	Gly12ValfsTer2	Homo	3/M	_	+	Bilateral severe to profound SNHL	85	Deafness, autosomal recessive 1A (220,290)	Rodriguez-Ballesteros et al. (2008)
8	*GJB2*	Gly12ValfsTer2	Compound hetero	4/F	+	_	Bilateral moderate SNHL	93	Deafness, autosomal recessive 1A (220,290)	Rodriguez-Ballesteros et al. (2008)
		Trp77Arg								Carrasquillo et al. (1997)
9	*BSND*	Gly47Arg	Homo	4/M	+	+	Bilateral severe to profound SNHL	87	Sensorineural deafness with mild renal dysfunction (602,522)	Miyamura et al. (2003)
10	*OTOF*	Glu747Ter	Homo	11/M	+	_	Bilateral severe to profound SNHL	90	Deafness, autosomal recessive 9 (601,071)	Rodriguez-Ballesteros et al. (2008)
11	*CDH23*	Asn1521Ser	Homo	4/F	+	_	Bilateral severe to profound SNHL	80	Deafness, autosomal recessive 12 (601,386)	Sloan-Heggen et al. (2015)
12	*TMIE*	Arg84Trp	Homo	6/F	+	+	Bilateral severe to profound SNHL	96	Deafness, autosomal recessive 6 (600,971)	Naz et al. (2002)
13	*SLC26A4*	p.?	Homo	3/F	+	+	Bilateral moderate SNHL	90	Deafness, autosomal recessive 4, with enlarged vestibular aqueduct (600,791)	Fugazzola et al. (2002)

Age is in years

*M* male, *F* female, *ABR* auditory brain stem response, *IQ* intelligence quotient, *OMIM* Online Mendelian Inheritance in Man, *N/A* not available

Whole exome sequencing of the 13 patients came up with conclusive results. Homozygous variants were detected in 11 patients, whereas compound heterozygous variants were detected in 2 patients. A total of 13 variants were detected in eight genes (*GJB2*, *MYO7A*, *MYO15A*, *BSND*, *OTOF*, *CDH23*, *SLC26A4*, and *TMIE*). The detected variants included one novel variant detected on the *MYO7A* gene and two variants first to be detected on the *OTOF* gene in patients with ARNSHL. Each of the two *OTOF* gene variants had very low allele frequency on the gnomAD (v4.1.0) database.

Different in silico functional prediction tools were used to predict the pathogenicity of the novel *MYO7A* gene variant as well as the two *OTOF* gene variants first to be detected in patients with ARNSHL. The tools supported the classification of the variants as likely pathogenic (Table 2). The 13 detected variants were subclassified into two splice variants, three nonsense variants, six missense variants, and two frameshift variants. The ACMG classification ranged from likely pathogenic to pathogenic as shown in Table 3.

**Table 2:** Variants detected for the first time in ARNSHL patients with different in silico prediction tools supporting their pathogenicity

In silico prediction engine	Variant		
	NM_000260.4(MYO7A): c.736-2A > C (Clinvar: SCV005184327)	NM_194248.3(OTOF): c.3704_3719dup (Clinvar: SCV005184329)	NM_194248.3(OTOF): c.1248G > A (Clinvar: SCV005184330)
dbscSNV^a^	Pathogenic strong (0.9999)	N/A	N/A
Splice AL^b^	Splice-altering/strong (0.98)	N/A	N/A
MaxEntScan^c^	Pathogenic strong (8.0423)	N/A	N/A
EIGEN^d^	Pathogenic moderate (0.9167)	N/A	Pathogenic moderate (0.9144)
GenoCanyon^e^	Deleterious (1)	N/A	Deleterious (1)
FATHMM-MKL^f^	Pathogenic moderate (0.9969)	N/A	Pathogenic supporting (0)
DANN^g^	Deleterious (0.98)	N/A	Deleterious (1)
EIGEN PC^h^	Pathogenic supporting (0.6833)	N/A	Pathogenic supporting (0.7609)
BayesDel noAF^i^	N/A	N/A	Pathogenic strong (0.6348)
BayesDel addAF^j^	N/A	N/A	Pathogenic strong (0.6075)
LRT^k^	N/A	N/A	N/A
CADD Score^l^ (V1.7)	32	N/A	39
MutationTaster^m^	Deleterious (1)	N/A	Deleterious (1)
Conservation Score PhyloP100^n^	8.926	6.746	7.848

*N/A* not available

^a^dbscSNV predicts splice site variants with a score ranging from 0 to 1 where higher scores are more deleterious

^b^Splice AL predicts the occurrence of splicing events with a score ranging from 0 to 1 where a higher score has a higher probability of being splice-altering

^c^MaxEntScan: the probability of being a true splice site sequence is given a higher score

^d^EIGEN score is a function prediction score for SNVs considering allele frequencies, conservation, and deleteriousness

^e^GenoCanyon predicts the functional potential of each position in the human genome using 22 experimental and computational annotations

^f^FATHMM-MKL: infers SNVs with scores higher than 0.5 to be deleterious

^g^DANN scores range from 0 to 1 the higher the score the more damaging effect is predicted

^h^EIGEN PC score is a function prediction score for SNVs considering allele frequencies, conservation, and deleteriousness

^i^BayesDel noAF scores range from − 1.31914 to 0.840878 where a higher score is more pathogenic

^j^BayesDel addAF scores range from − 1.11707 to 0.750927 where higher scores indicate that the variant is more likely to be pathogenic

^k^Likelihood ratio test (LRT) predicts the deleteriousness of variants through the detection of highly conserved amino acid regions within a set of 32 vertebrate species the scores range from 0 to 1

^l^Combined Annotation Dependent Depletion (CADD) scores are a tool for scoring the deleteriousness of SNVs in the human genome where higher scores indicate more deleterious variants

^m^MutationTaster predicts the potential of a variant to cause disease. The score ranges from 0 to 1 the higher scores indicate more deleterious variants

^n^PhyloP100 score is a conservation score through multiple alignments of 99 vertebrate genome sequences to the human genome. The higher the score, the more conserved the site

**Table 3:** Molecular results of all patients with ARNSHL enrolled in the study

Patient	Gene	Transcript	Nucleotide change	Protein change	Variant type	ACMG classification (criteria)	REVEL	Allele frequency (gnomAD v4.1.0)	Splice AL
P1	*MYO7A*	NM_000260.4	c.736-2A > C^a^	p.(?)	Splice acceptor	Likely pathogenic (PVS1, PM2)	N/A	Not found	0.98 (strong)
P2	*MYO7A*	NM_000260.4	c.3997C > T	p. Gln1333Ter	Nonsense	Likely pathogenic (PVS1, PM2)	N/A	Not found	N/A
P3	*OTOF*	NM_194248.3	c.3704_3719dup^a^	p. Ser1240ArgfsTer57	Frameshift	Likely pathogenic (PVS1, PM2)	N/A	0.000000685	N/A
P4	*OTOF*	NM_194248.3	c.1248G > A^a^	p. Trp416Ter	Nonsense	Likely pathogenic (PVS1, PM2)	N/A	0.000000684	N/A
P4	*OTOF*	NM_194248.3	c.5374C > T	p. Arg1792Cys	Missense	Pathogenic (PM3, PM2, PM5, PP3, PM1, PP5)	0.84	0.000009913	N/A
P5	*MYO15A*	NM_016239.4	c.5203C > T	p. Arg1735Trp	Missense	Likely pathogenic (PP5, PM2, PP3)	0.76	0.000003098	N/A
P6, P7, P8	*GJB2*	NM_004004.6	c.35delG	p. Gly12ValfsTer2	Frameshift	Likely pathogenic (PVS1, PM2, PP5)	N/A	0.007050	N/A
P8	*GJB2*	NM_004004.6	c.229T > C	p. Trp77Arg	Missense	Pathogenic (PM3, PM2, PM1, PP3, PS3, PP2, PP1, PP5)	0.93	0.00003531	N/A
P9	*BSND*	NM_057176.3	c.139G > A	p. Gly47Arg	Missense	Pathogenic (PM3, PM2, PS3, PP1, PP5)	0.5	0.0001897	N/A
P10	*OTOF*	NM_057176.3	c.139G > A	p. Glu747Ter	Nonsense	Pathogenic (PM3, PVS1, PM2, PP5)	N/A	0.000001860	N/A
P11	*CDH23*	NM_022124.6	c.4562A > G	p. Asn1521Ser	Missense	Likely pathogenic (PP3, PM3, PM2, PP5)	0.83	0.00001301	N/A
P12	*TMIE*	NM_147196.3	c. 250C > T	p. Arg84Trp	Missense	Likely pathogenic (PP3, PM3, PM2, PP5, PM5)	0.73	0.00001859	N/A
P13	*SLC26A4*	NM_000441.2	c.1614 + 1G > A	p.(?)	Splice donor	Pathogenic (PM3, PVS1, PM2, PP5)	N/A	0.00002765	1 (strong)

*N/A* not available

^a^Variants reported for the first time in patients with ARNSHL. REVEL is an ensemble method for the prediction of pathogenicity of missense variants based on 13 individual tools: MutPred, FATHMM, VEST, PolyPhen, SIFT, PROVEAN, MutationAssessor, MutationTaster, LRT, GERP, SiPhy, phyloP, and phastCons. The REVEL score ranges from 0 to 1 for an individual missense variant, where variants with a higher probability of causing disease have higher scores. Splice AL predicts the occurrence of splicing events with a score ranging from 0 to 1 where a higher score has a higher probability of being splice-altering

**Fig. 1:**
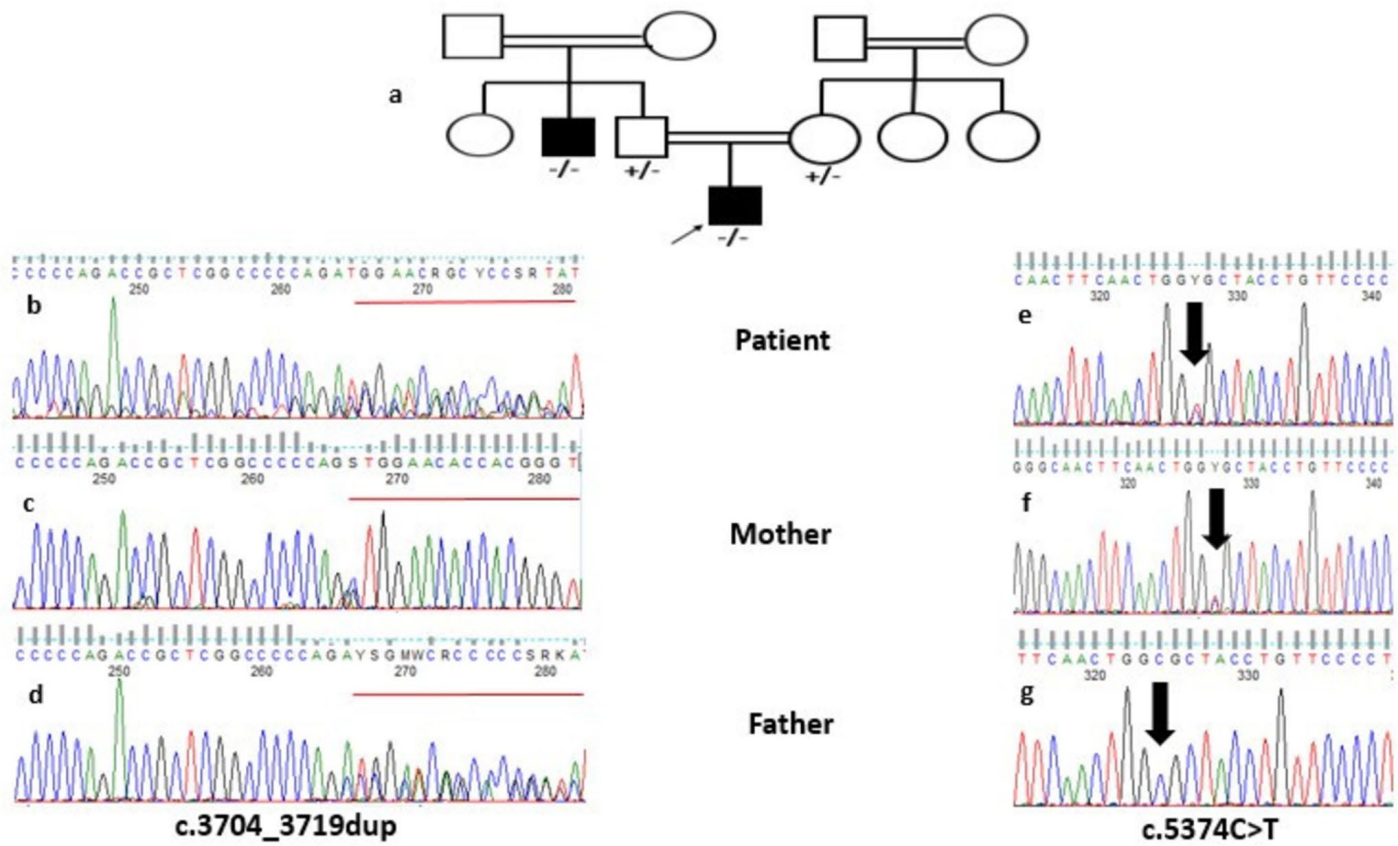
Three generations family pedigree for patient 4 (a). Electropherograms showing familial segregation of variant c.3704_3719dup in the *OTOF* gene where patient 4 (b) and the father (d) show heterozygous form, while the mother shows wild type (c). Electropherograms showing familial segregation of variant c.5374C > T in the *OTOF* gene where patient 4 (e) and the mother (f) show heterozygous form, while the father shows wild type (g)

Sanger sequencing confirmation and segregation were done for all available family members in the 13 families (Fig. 1 Supplementary). Segregation was of special importance to P4 who carried compound heterozygous variants in the *OTOF* gene. The two variants were confirmed in the patient on trans alleles. The previously reported missense variant was inherited from the mother (carrier for c.1248G > A). The frameshift 16 base pair (bp) duplication, first time to be detected in a patient with ARNSHL, was inherited from the father (carrier for c.3704_3719dup) as shown in Fig. 1.

## Discussion

Non-syndromic sensorineural hearing loss (NSHL) is 75–80% inherited in an autosomal recessive pattern (Vona et al. 2015). ARNSHL has a relatively high prevalence in Egypt (Elbagoury et al. 2022). This can be attributed to the elevated rate of parental consanguinity (Temtamy and Aglan 2012). Genetic sensorineural HL is mainly caused by pathogenic variants in genes expressed in the inner ear where the cochlea plays an important role in transmitting the sound waves through a cascade of hair cells’ depolarization-repolarization reactions through influx and efflux of potassium and calcium ions. This in turn triggers neurotransmitter release activating the acoustic nerve (Morgan et al. 2020, Willems 2000).

The inner ear’s gene expression patterns are becoming more understood, which helped in multitherapy intervention. More than 140 genes have been reported to be expressed in the inner ear. They are involved in one way or another in the hearing mechanism (Delmaghani and El-Amraoui 2020).

Our study is an attempt to investigate the genetic etiology of ARNSHL through the recruitment of 13 patients from 13 unrelated families followed by carrying out whole exome sequencing to detect the pathogenic gene variants responsible for HL in those patients.

The molecular analysis revealed the presence of 13 variants in 8 genes among the 13 patients. Three variants first to be reported in patients with ARNSHL (one nonsense, one splice site, and one frameshift) were detected in three patients. Ten previously reported variants were detected in the remaining patients.

The eight genes in which variants were detected in this study can be classified according to their function into three groups. *MYO7A*, *TMIE*, *CDH23*, and *MYO15A* are involved in hair bundle development and function. *GJB2*, *BSND*, and *SLC26A4* are involved in Cochlear ion homeostasis. The *OTOF* gene is involved in synaptic transmission (Delmaghani and El-Amraoui 2020).

A splice site variant located in the canonical region of intron 14 in the *MYO7A* gene (c.736-2A > C) was detected in a homozygous form in P1. The splicing error seems to be a common mechanism of the disease in this gene since about 78 splice site variants have been reported in the *MYO7A* gene so far (HGMD database accessed 12 August 2024). The variant is predicted to cause alteration in splicing which might lead to the inclusion of intron or exclusion of exon resulting in an improper transcription and consequently production of aberrant mRNA liable to nonsense-mediated mRNA decay or production of nonfunctioning protein.

Two nonsense variants, namely p. Gln1333Ter and p. Trp416Ter, were detected in *MYO7A* and *OTOF* genes, respectively. The *MYO7A* variant detected in P2 causes the production of a truncated 1333 amino acid (aa) protein instead of the 2215 aa long protein. This variant was reported once before in an Egyptian family (Budde et al. 2020). The variant in the *OTOF* gene detected in P3 yields a protein 416 aa long instead of the 1997 aa long protein. Each of the two variants may lead to a nonsense-mediated decay which could abolish the transcripts produced.

A 16 bp duplication (c.3704_3719dup) was detected for the first time in a patient with ARNSHL in this study. The variant was detected on one allele of the *OTOF* gene in P4 in compound heterozygosity with a reported missense variant in the same gene. The duplication frameshift variant resulted in the production of a truncated protein 1297 aa long instead of 1997 aa long normal protein. The variant had a predicted ACMG classification of *likely pathogenic*. The *OTOF* gene has Clingen specific guidelines highlighting that null variants including frameshift variants in this gene where the loss of function (LOF) is a known mechanism of disease have strong evidence of pathogenicity. The variant has a gnomAD (v4.1.0) frequency of 0.000000685 (allele count = 1/1460074) which supports the hypothesis that it is a variant that can cause HL in recessive form. Familial segregation for this patient verified that the inheritance of the detected variants was from both parents not in trans from one parent.

Interestingly, P9 carried a homozygous variant in the *BSND* gene (p. Gly47Arg). This variant is known to cause sensorineural deafness with mild renal dysfunction also known as Bartter syndrome. This variation was initially identified in 2003 in a patient who, at the age of 28, had mild renal dysfunction and SNHL. The primary complaint in our 4-year-old patient was bilateral severe to profound SNHL, leading to a provisional classification of non-syndromic HL. When the parents were questioned about any complaints regarding the kidneys, they stated that he had a burning sensation during urination. This may be consistent with published reports of SNHL patients experiencing delayed, mild renal complaints, describing the condition as atypical Bartter syndrome (Miyamura et al. 2003).

The majority of the patients studied in this cohort suffered severe to profound bilateral hearing loss except for two patients who showed moderate hearing loss (P8 and P13). P8 carried compound heterozygous variants (p. Gly12ValfsTer2/p. Trp77Arg) in the *GJB2* gene where the p. Trp77Arg variant bearing allele seems to have some residual protein activity partially counteracting the loss of function protein produced by the p. Gly12ValfsTer2 variant bearing allele. P13 carried a homozygous splicing variant (c.1614 + 1G > A) in the *SLC26A4* gene which also seems to produce a protein with some residual function impacting the degree of hearing loss.

In Egypt, most of the studies done on ARNSHL focused on the *DFNB1* locus which encompasses two genes namely *GJB2* and *GJB6*. The first study was done in 2005 for the detection of mutations in *GJB2* as well as detecting del (*GJB6*-D13S1830) in the *GJB6* gene in a cohort of 159 Egyptian patients from 111 families and revealed the absence of del (*GJB6*-D13S1830) as well as the presence of six different variants in *GJB2* gene where c.35delG was the most common variant. The other five variants were p.Thr8Met, p.Val37Ile, p.Val153Ile, c.333_334delAA, and IVS1 + 1G > A (Snoeckx et al. 2005). This study was followed by another one which detected c.35delG in 10.17% of patients with ARNSHL enrolled (Meguid et al. 2008). In 2014, two studies were published: one was concerned with detecting mutations in *GJB2* as well as detecting presence of del (*GJB6*-D13S1854) and del (*GJB6*-D13S1830) in *GJB6* gene in a cohort of 36 patients where the allelic frequency of c.35delG in *GJB2* gene was 18%, whereas no deletions were detected in *GJB6* gene (Elbagoury et al. 2014). The other study investigated mitochondrial hearing loss. It was conducted on 97 patients to detect the mitochondrial 1555A > G variant in the *MTRNR1* gene. The variant was found with a frequency of 1.3% (Fassad et al. 2014). A fourth study was conducted on 51 patients mainly concerned with the detection of c.35delG and c.167delT in the *GJB2* gene. It revealed the absence of c.167delT and the presence of c.35delG with an allelic frequency of 10.8% (El Barbary et al. 2015). A more comprehensive study done on 61 consanguineous Egyptian families using the WES technique came up with the detection of variants in 23 different genes. The majority of variants were located in *MYO15A*, *SLC26A4*, *GJB2*, and *MYO7A* (Budde et al. 2020).

*OTOF* and *GJB2* gene variants represented the majority (50%) of the detected variants in our cohort. *GJB2* gene has been the most common cause of ARNSHL in many populations specifically the c.35delG variant which is responsible for almost 63% of cases in North America, Europe, and the Middle East (Azadegan‐Dehkordi et al., 2019). On the other hand, many studies conducted in diverse regions attributed ARNSHL in 2.3 to 7.3% of cases to variants in the *OTOF* gene (Duman et al. 2011; Iwasa et al. 2013). In the Saudi Arabian population, the *OTOF* gene variants are considered significant contributors to ARNSHL (Almontashiri et al. 2018). Remarkably, *GJB2* and *OTOF* have been targets for plenty of pre-clinical gene therapy projects. On the other hand, some approaches are concerned with inner ear hair cell regeneration regardless of the genetic cause using the Hath1 transcription factor (Isherwood et al. 2021).

In conclusion, this study added the *OTOF* gene to the list of the most common genes causing ARNSHL among Egyptian patients. Three variants in the *MYO7A* and *OTOF* genes were detected for the first time in patients with ARNSHL broadening their genetic spectrum. It highlights how next-generation sequencing (NGS) technology can be applied to accurately detect patients with non-classical syndromic deafness misdiagnosed as non-syndromic deafness. It emphasizes the importance of detecting the gene involved in the pathogenesis of non-syndromic deafness for each patient as a step towards tailored gene therapy. However, a larger cohort of patients should be recruited in future studies. *GJB2* gene variants could be ruled out through Sanger sequencing as a cost-saving step before proceeding to WES in any future study. Implementation of experimental functional analysis is highly recommended to give a more comprehensive genetic background about ARNSHL in Egypt.

## Supplementary Information

Below is the link to the electronic supplementary material.

Supplementary file1 Family Pedigree for the 13 studied patients with ARNSHL. Segregation of the variants is presented on the pedigree as +/+, +/- and -/- (+ for normal allele and – for mutated allele). F: Family, P: Patient (JPG 171 KB)

## Data Availability

No datasets were generated or analyzed during the current study.
