# Supplementary figures and images for: Molecular and Clinical Characterization of a Cohort of Autosomal Recessive Sensorineural Hearing Loss in Egyptian Patients
Source: J Mol Neurosci. 2024 Oct 28;74(4):102. doi: 10.1007/s12031-024-02279-3 (PMC11519120; doi:10.1007/s12031-024-02279-3)

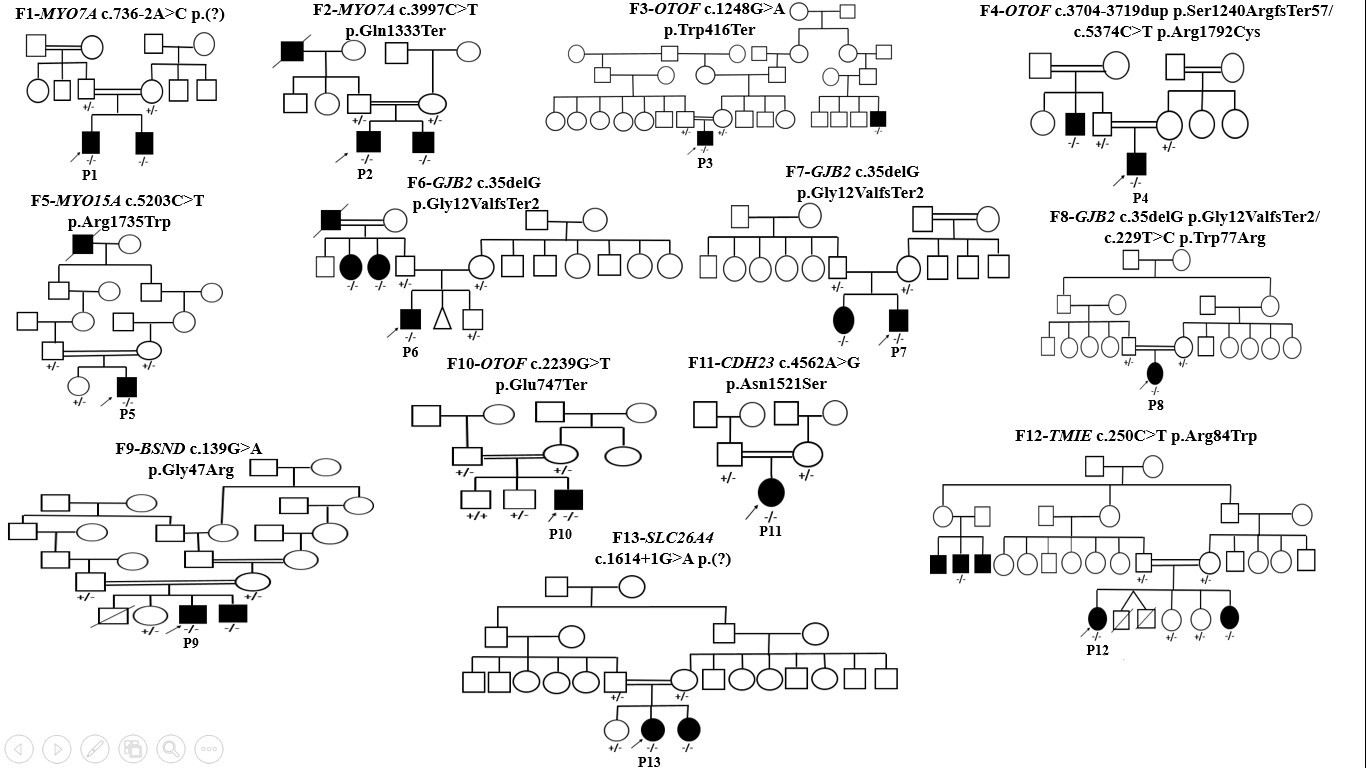

Supplement: Supplementary file 1 — Supplementary file1 Family Pedigree for the 13 studied patients with ARNSHL. Segregation of the variants is presented on the pedigree as +/+, +/- and -/- (+ for normal allele and – for mutated allele). F: Family, P: Patient (JPG 171 KB) [file 12031_2024_2279_MOESM1_ESM.jpg]
